# Treatment of Advanced Carcinoma of the Larynx and Hypopharynx with Laser Followed by External Radiotherapy

**Published:** 2017-09

**Authors:** Lalee Varghese, John Mathew, Subhashini John, Anand Job

**Affiliations:** 1 *Department of Otorhinolaryngology, Christian Medical College, Vellore, India.*; 2 *Department of Radiotherapy, Christian Medical College, Vellore, India.*

**Keywords:** Hypopharynx, Laser debulking, Larynx, Malignancy, Tracheostomy

## Abstract

**Introduction:**

Radical laryngeal surgeries for extensive laryngeal and hypopharyngeal tumors often require a permanent tracheostomy, which has an immense impact on the quality of life of patients. A minimally invasive technique such as transoral laser microresection (TLM) followed by radiotherapy can preserve the functions of the voice and swallowing. The aim of this study is to evaluate the role of laser debulking in the treatment of carcinoma of the larynx and hypopharynx, to evaluate the response of the tumor to subsequent radiotherapy, and also to assess the usefulness of laser in avoiding tracheostomy and functional preservation of the voice and swallowing.

**Materials and Methods:**

This prospective cohort study included patients with carcinoma of the larynx and hypopharynx unwilling to have definitive surgery and those medically unfit for radical surgery. The clinical profile of patients at presentation, tumor status following laser debulking, immediately after radiotherapy (RT), 6 weeks post RT, 3 months post RT, and at the end of study; short term complications associated with laser surgery; and usefulness of laser in avoiding tracheostomy and in functional preservation of the voice were evaluated.

**Results:**

There were 18 (90%) male patients and 2 (10%) female patients. Age ranged from 24 to 78 years with a mean age of 55. Hoarseness of voice was the most frequent presenting complaint (90%) followed by progressive dysphagia (45%), odynophagia (40%), otalgia (40%), and dyspnoea (25%). 11 (55%) patients had T3 tumors, while 6 (30%) were T2, and 3 (15%) were T4 lesions. 65% of patients were free of lymph node metastasis at presentation. 2 (10%) had N1 and 5 (25%) had N2 nodes. At presentation 10 (50%) patients had Stage III disease and 6 (30%) had stage IV disease. 13 patients (65%) had moderately differentiated squamous cell carcinoma. None of the risk factors and co-morbid illnesses showed any statistically significant difference among the tumor sites. Apart from the 2 (10%) patients who had residual disease, 2 (10%) patients developed a recurrent tumor in the course of their follow up. None had neck recurrence. Two patients underwent tracheostomy, before laser surgery, for compromised airway and both had recurrence of their tumor and continued to be on tracheostomy.

**Conclusion:**

Laser debulking followed by radiotherapy is a viable alternative in the management of malignancies of the larynx and hypopharynx for those who refuse radical surgery and for those patients in whom radical open surgery is impractical due to physiological reasons such as advanced age and poor pulmonary reserve.

## Introduction

The larynx is the second most common site of primary epithelial malignant tumors in the head and neck region, and represents approximately 2% of total cancer risk (1). In the management of extensive laryngeal and hypopharygeal tumors, radical surgery, when performed, often requires a permanent tracheostomy. Considering the key role of the larynx in phonation, respiration, and deglutition, cancer of this organ and its management has an immense impact on the quality of life of the patient. Organ preservation surgery has been evolving to resect the tumor through an endoscope and to preserve the laryngeal function. The development of transoral laser microsurgery (TLM), a relatively new conservative surgical technique, using a carbon dioxide laser via an endoscopic approach is found to be one of the most useful procedures. 

The purpose of this study is to determine a potential alternative to radical surgery for laryngeal and hypopharyngeal cancers, which may also help avoid a tracheostomy prior to or during radiotherapy. We have evaluated the role of a minimally invasive technique of resection of advanced laryngeal tumors. This technique uses a laser through an endoscope under magnification (using an operative microscope) followed by radiotherapy in patients with carcinoma of the larynx and hypopharynx who are unwilling to have definitive surgery and those medically unfit for radical surgery.

## Materials and Methods

Patients with T2, T3 & T4 carcinomas of the larynx and hypopharynx who were unwilling to have definitive surgery and those medically unfit for radical surgery were included in the study. Patients with distant metastasis and those who had undergone previous treatment with radiotherapy, chemotherapy or surgery were excluded from the study. Approval was obtained from the Institutional Review Board prior to starting the study. After obtaining a written informed consent from the patients, they were treated with microendoscopic laser surgery followed by external radiotherapy. Staging of the patients was done using the International Union against Cancer (UICC) staging system. Of the 20 patients included in the study, 10 (50%) had Stage III disease and 6 (30%) had stage IV disease at presentation. 

*Technique*

All the patients had a histopathological confirmation of the diagnosis of malignancy prior to therapy. The patients had laser resection or debulking of the primary tumor followed by postoperative radiotherapy (RT) to the primary tumor and neck. All transoral resections and debulking of the tumor were performed using laser and were carried out under general anaesthesia and endotracheal tubes with cuff protected by a double layer of saline-saturated cottonoids or flexible metallic tubes were used. Optimum exposure and visualization of the larynx and the hypopharynx, which is crucial for a safe tumor resection, was always ensured. Different laryngoscopes, including bivalved adjustable laryngoscopes were used to expose the larynx.

A carbon dioxide laser was used, coupled with Zeiss operating microscope with a 400mm lens allowing precise co-axial delivery of both the helium neon aiming beam and CO2 cutting beam to the operating field. The focus diameter of the micromanipulator was 0.5mm and the delivered laser energy was about 5-10 watts continuous mode. Since the CO2 laser coagulates vessels with a maximum diameter of 0.5 to 1mm, any bleeding from large vessels was controlled by electrocautery.

A perioperative antibiotic prophylaxis, usually with amoxycillin, was administered. In addition, an intraoperative dose of corticosteroids was administered in more extended resections to decrease the likelihood for laryngeal oedema. 

After laser resection or debulking, radical radiotherapy was administered to the primary tumor and draining neck nodes. This was indicated with a radical or prophylactic dose, taking into account the high risk for occult sub clinical nodes even in the absence of palpable lymph nodes. Postoperative radiotherapy was performed with photons with conventional 2 dimensional radiotherapy technique using Cobalt - 60 beam. A radical dose of 66Gy was given to the primary and involved neck nodes and a prophylactic dose of 50Gy was given for clinically uninvolved neck nodes, at a daily dose of 1.8 to 2Gy. Appropriate shielding of the spinal cord was performed at 40Gy and the remaining dose of 10Gy to the posterior neck was delivered using electrons. The patients did not receive any other treatment until local recurrence or metastases appeared. All patients were evaluated at fixed intervals to assess the tumor status:- post laser excision just prior to RT, mid RT, end of RT, 6 weeks post RT and 3 months post RT. Long term follow up was also carried out to assess the outcome. Local control was measured from initiation of therapy to the most recent follow-up or local failure. During follow-up, the larynx was clinically assessed by indirect laryngoscopy and fibreoptic laryngoscopy. Repeated microlaryngoscopy and excisional biopsies were performed when relapses were suspected. Laser surgery related complications like pneumonia (2), haemorrhage, dysphonia, persistent dysphagia, laryngeal chondritis, perioperative bronchospasm, and cutaneous burns, if any, were noted in addition to the usefulness of laser in the preservation of swallowing and in reducing morbidity by avoiding tracheostomy. 

## Results

Twenty subjects were enrolled in this cohort study to evaluate the treatment outcome after laser resection or debulking and external radiotherapy in the management of carcinoma of the larynx and hypopharynx. All the data was entered in a standard proforma and analyzed in the SPSS program. There was a significant male predominance with 18 (90%) male and 2 (10%) female patients in the study group. Age ranged from 24 to 78 years with a mean age of 55. Among these patients 10 (50%) were in the 60-80 age group, 7 (35%) in the 40-60 age group, and the other 3 in the 20-40 age group. Among the clinical features at initial presentation, hoarseness of voice was the most frequent (90%) followed by progressive dysphagia (45%), odynophagia (40%), otalgia (40%) and dyspnoea (25%) (Table.1).

**Table.1:** Symptoms at presentation vs Tumour location

	**Supraglottic**	**Glottic**	**Transglottic**	**Laryngo-pharynx**	**Total Numbers**	**Percentage**
Hoarseness of voice Breathing difficulty Noisy breathing Progressive difficulty in swallowing Odynophagia Earache Neck pain Neck swelling Cough Hemoptysis Anorexia Weight loss	3 0 0 1 2 3 1 0 0 0 0 1	5 0 0 2 0 0 0 0 1 0 0 0	4 3 3 0 0 2 0 0 1 1 0 0	6 2 1 6 6 3 3 3 3 2 1 2	18 5 4 9 8 8 4 3 5 3 1 3	90 25 20 45 40 40 20 15 25 15 5 15
						

Among the 20 patients, 15 (75%) were chronic smokers and 11 (55%) had habit of tobacco chewing (Table.2). 

**Table 2:** Risk factors and co-morbid illnesses

	**Numbers**	**Percentage**
Smoking Tobacco chewing Hypertension Diabetes mellitus	15 11 4 5	75 55 20 25
		

Four (20%) of the patients had hypertension and 5 (25%) had diabetes mellitus. Of the twenty patients, 4 had supraglottic, 5 glottic, 4 transglottic and 7 laryngopharynx carcinomas. The majority of patients ie 11 (55%) had T3 tumors, while 6 (30%) had T2 and 3 (15%) had T4 lesions and 65 % of patients were free of lymph node metastasis. Among the patients who had metastasis to the lymph nodes, 2 (10%) had N1 and 5 (25%) had N2 nodes (Table.3).

**Table 3:** Tumour location vs Tumour T status and Nodal N status

	**T2**	**T3**	**T4**	**No**	N1	N2	Total Nos	Total %
Supraglottic	0	4	0	4	0	0	4	20%
Glottic	4	1	0	4	1	0	5	25%
Transglottic	0	3	1	3	1	0	4	20%
Laryngopharynx	2	3	2	2	0	5	7	35%
Total	6 (30%)	11 (55%)	3 (15%)	13(65%)	2(10%)	5(25%)		

Upon presentation, 10 (50%) patients had Stage III disease, 6 (30%) had stage IV disease, and 4 (20%) had stage II disease. Histopathological examination showed that the majority had moderately differentiated squamous cell carcinoma (13 patients, 65%) followed by well differentiated squamous cell carcinoma (3 patients,15%) and poorly differentiated squamous cell carcinoma (4 patients, 20%). 

There was no statistically significant difference among the risk factors and co-morbid illnesses among the tumor sites. Complications of laser surgery like pneumonia, dysphonia, persistent dysphagia, laryngeal chondritis, perioperative bronchospasm, cutaneous burns and ignition of ETT were absent in all 20 patients. There was excessive peroperative bleeding in 1 (5%) patient. 

Following laser excision, 18 (90%) patients had no evidence of a gross tumor and continued to be disease free at the 3 month post radiotherapy follow up and at their last follow up which ranged from 4 months to 9 years 10 months post therapy, with a mean of 3 years and 81 days. The duration of the follow up was more than 5 years in 6 patients and more than 1 year in 5 patients. 

Apart from the 2 patients who had residual disease post laser excision, upon follow up, 2 more patients developed a recurrent tumor in the primary site at 13 months and 19 months post treatment while none of the other patients had residual or recurrence in the neck. 

## Discussion

The recommended treatment for patients with extensive laryngeal and hypopharyngeal tumors (T3& T4) is radical surgery such as total laryngectomy with or without pharyngectomy requiring a permanent tracheostomy and voice restoration with valve prosthesis, electro larynx or other methods such as oesophageal speech. Voice restoration is the most important therapeutic challenge in the rehabilitation of these patients. Depriving the patient of the ability to communicate verbally is the greatest difficulty, which can strongly affect a head and neck cancer patient, at times making him refuse radical surgery and opt for larynx-sparing procedures. Also some patients are unwilling to obtain a permanent tracheostomy. Our purpose in conducting this study was to determine a potential alternative to radical surgery for laryngeal and hypopharyngeal cancers.

For local disease control among patients with head and neck cancer treated with radiation therapy, tumor volume is a more important prognostic factor than T and N stage and site of disease. In a study by Doweck et al, patients with tumor volumes smaller than 19.6cc had 93.8% local control, compared with 57% for the patients with a volume greater than 19.6cc (3). Biologically, this can be explained by tumor hypoxia (4). Large, bulky lesions typically have areas of central tumor necrosis, which may be extensive. It is difficult to deliver chemotherapy agents effectively to such massive tumors that have outstretched their blood supply. For advanced disease, primary radical radiotherapy alone (single modality) has been well known to yield unfavorable results, with 5-year survival rates reported ranging from 5% to 30%. Hence, in current practice, combining surgical treatment and radiotherapy has a solid rationale. In this study, we evaluated the outcomes of laser surgery and also weighed the benefits of voice preservation.

All of the 20 patients included in the study declined definitive surgery, wanted to avoid laryngectomy, and favored function-preserving management. In view of this, since the patients had locally advanced disease, a combined modality approach of debulking of the tumor using microendoscopic laser surgery instead of radical surgery was carried out followed by external beam radiotherapy. Transoral laser microresection in usually used for surgical resection with a curative intent. In our study, we performed a resection of advanced laryngeal tumors using a laser through an endoscope under magnification by using an operative microscope. We have used this minimally invasive technique to reduce the tumor bulk in advanced cancers. The proposed theory is that a debulked cancer with less tumor volume is more likely to be cured with radiation than the original larger tumor which may have islands of devascularised tissue. In addition, a permanent tracheostomy could be avoided. 

Zeitels reported 23 supraglottic cancers treated endoscopically (5). In 16 patients, tumor free margins were obtained. All were subsequently irradiated and there were no local recurrences. The 7 other patients with positive margins were also irradiated. Among these patients there was a higher incidence of local failure. 

 In our study, patients’ age ranged from 24 to 78 years with a mean age of 55. This was consistent with other reports (6). The male-to-female ratio of 9:1 may be reflective of differences in tobacco and alcohol use.

In the study population, 16 (80%) had stage III or IV disease. The 5-year survival rates of patients with hypopharyngeal carcinomas of all tumor stages vary between 14% and 28% and rarely exceed 30% regardless of the treatment approach used (7). As radical surgery and postoperative radiotherapy could not improve the locoregional control rate and roughly one third of the patients die as a result of distant metastases, second primary cancers, and intercurrent diseases, it seems judicious to consider organ preservation therapy regimen to improve at least the quality of life in patients with a known unfavorable prognosis. 

Since its introduction by Strong and Jako in 1972, CO_2_ laser has found a wide spectrum of applications in the treatment of laryngeal diseases (8). Its advantages^1^ include the expeditious nature of laser surgery with the duration of hospitalization of only few days. In addition, pain is minimal and the patient begins oral feeding on the second day after surgery with early recovery of normal swallowing. Also, an adequate airway can be provided and tracheostomy avoided, thus providing a much better unaltered quality of life. Each margin of the cancer is accessible to the operator’s view, magnified and illuminated by the microscope, from the beginning to the end of the surgery. 

Chances of imposing excessive excision are minimized and each excision can be adapted individually to the size of the lesion. The CO_2_ laser coagulates vessels up to a maximum diameter of 0.5 to 1mm, making the surgical field bloodless thus aiding precise and safe resection of the tumor. The laser seals nerve endings and lymphatics preventing micrometastasis by lymphatics and blood stream and postoperative pain and oedema are less (9). A laser beam is a light scalpel and there is no physical contact during tumor excision (10). So there is no risk of transferring cancer cells to another location. Hence it is permissible (with a CO_2_ laser) to subdivide laryngeal cancer in order to to facilitate better exposure and excise the tumor completely (11,12). Since transoral laser microresection approaches carcinomas through the mouth, requiring no disassembly of the neck, the local microvasculature continues undisturbed and there is no risk of pharyngocutaneous fistula. After laser excision, all other treatment options can still be used. If the lesion recurs or if a neoplastic lesion subsequently appears, not only can laser resection be repeated but other suitable alternative treatment options are also still available.

The two patients in the study who had residual disease at the primary site following laser surgery also continued to have the disease after radiotherapy and were given palliative chemotherapy. Two (10%) had “true” local recurrence, that is, reappearance of the cancer in the primary site after all initial therapy was completed at 13 months and 19 months respectively (Table.4). 

**Table 4:** Recurrent tumour

**Initial involved structures**	**Site of Recurrence**	**Time in Months**	**Treatment** ** Given**
Left false cord, vocal cord, both ventricles, both arytenoids and subglottis	Left false cord and ventricle	13 months	Laser excision of tumour and palliative chemotherapy
Right true cord, false cord, ventricle, subglottis and left vocal cord	Right arytenoid, both AEFs, base of epiglottis	19 months	Laser excision of tumour and total laryngectomy
			

All the patients including the seven patients who had a clinically palpable neck node at presentation were free of neck disease after radiotherapy and no neck recurrence was observed even until the last follow-up in these patients. 

In a study by Steiner on laser resection of T1 and T2 glottic tumors (12), the local recurrence rate was 8%. Rudert et al, in a study on laser resections of supraglottic tumors, had a recurrence rate of 13%. In addition, in Steiner’s study on pyriform sinus carcinoma, 9.3% of patients developed local recurrences and 3.9% of patients had locoregional recurrences (13). In our study the local recurrence rate was 10% and was observed after an interval of 13 months and 19 months after primary treatment. Both the patients who developed local recurrence at the primary site had stage 4 disease upon initial presentation and had to undergo an emergency tracheostomy for a compromised airway, before the laser surgery and were unable to undergo decannulation until the last follow up. They were treated with a curative intent by further transoral laser microsurgical resection and one of them also received palliative chemotherapy.

In a study by Herchenhorn et al on the impact of previous tracheotomy as a prognostic factor in patients with locally advanced squamous cell carcinoma of the larynx submitted to concomitant chemotherapy and radiation, patients who had previous tracheotomy, in comparison to those without a tracheotomy, had a lower rate of complete response (41.7 vs. 75%), shorter progression free-survival and median overall survival (12 vs. 56 months). Moreover a significant difference was observed in the 3-year survival rates (6 vs. 61%) (14). In a review of transglottic carcinoma (15), patients who required a pretreatment tracheostomy had a lower prognosis than those who did not and stomal disease developed in many of the former patients.

None of our patients had any laser related complications except one patient (5%) who had excessive bleeding, which was controlled by external carotid artery ligation. Although an objective voice analyses was not done, all of our patients had intelligible speech at the last follow up.

## Conclusion

In view of the good local control and feasibility of organ preservation, laser debulking followed by radiotherapy is a viable alternative in the management of malignancies of the larynx and hypopharynx for those who are unwilling to undergo radical surgery and for those patients in whom radical open surgery is impractical due to physiological reasons such as advanced age and poor pulmonary reserve. The advantage of laser is that by reducing tumor volume, better cure rates can be achieved by subsequent radiotherapy. In addition, there is functional preservation of swallowing and speech and also tracheostomy can be avoided. Our results show an encouraging trend towards adequate disease control following organ-preserving and function-preserving laser surgery followed by radiotherapy. However a larger prospective study will be required to establish the effectiveness and benefit of this new therapeutic concept. 
